# Development and validation of the Lebanese Social Media Dependency Scale (LSMDS): A cross-sectional study among university students

**DOI:** 10.1371/journal.pone.0344535

**Published:** 2026-04-28

**Authors:** Samar Younes, Zeinab Abbas, Chadia Haddad, Daniele Saade, Nisreen Mourad, Hala Sacre, Amal Al-Hajje, Pascale Salameh

**Affiliations:** 1 School of Pharmacy, Lebanese International University, Bekaa, Lebanon; 2 INSPECT-LB (Institut National de Santé Publique, d’Épidémiologie Clinique et de Toxicologie-Liban), Beirut, Lebanon; 3 Inserm U1094, IRD UMR270, Univ. Limoges, CHU Limoges, EpiMaCT - Epidemiology of chronic diseases in tropical zone, Institute of Epidemiology and Global Health – Michel Dumas, OmegaHealth, Limoges, France; 4 Gilbert and Rose-Marie Chagoury School of Medicine, Lebanese American University, Beirut, Lebanon; 5 Ministry of Public Health, Narcotics Department, Beirut, Lebanon; 6 Research Department, Psychiatric Hospital of the Cross, Jal-Eddib, Lebanon; 7 Faculty of Public Health, Lebanese University, Fanar, Lebanon; 8 IVPN-Network, Fujairah, United Arab Emirates; 9 Drug Information Center, Order of Pharmacists of Lebanon, Beirut, Lebanon; 10 Faculty of Pharmacy, Lebanese University, Hadath, Lebanon; 11 School of Medicine, Lebanese American University, Beirut, Lebanon; 12 Department of Primary Care and Population Health, University of Nicosia Medical School, Nicosia, Cyprus; University of Hafr Al-Batin, SAUDI ARABIA

## Abstract

**Background:**

Social media use has become pervasive among university students worldwide, with growing evidence linking excessive use to negative psychological and academic outcomes. While various scales exist to assess problematic social media use, most have been validated in Western populations, limiting their cultural applicability. This study aimed to develop and validate a culturally appropriate Lebanese Social Media Dependency Scale (LSMDS) for use among university students in Lebanon.

**Methods:**

A cross-sectional study was conducted from March to April 2025 among 511 university students across Lebanon. The LSMDS was developed by selecting and refining items from three existing scales: the Smartphone Addiction Inventory (SPAI), Online Fear of Missing Out Inventory (ON-FoMO), and Social Media Disorder Scale (SMD). Exploratory factor analysis with oblimin rotation was performed to determine the factor structure. Construct and convergent validity were assessed, along with reliability testing using Cronbach’s alpha coefficients.

**Results:**

The final LSMDS comprised 27 items organized into three factors explaining 52.91% of total variance: Problematic Smartphone Use (11 items), Social Media Validation Seeking (8 items), and Social Media Withdrawal Symptoms (8 items). The scale demonstrated excellent internal consistency (Cronbach’s α = 0.931) with strong reliability for individual factors (α = 0.847–0.913). Convergent validity was supported by significant correlations with SPAI (r = 0.863) and ON-FoMO (r = 0.888), and moderate correlation with SMD (r = 0.621). Single participants showed significantly higher LSMDS scores compared to married participants (47.50 vs 39.33, p = 0.037). Multivariable analysis revealed that a higher score on the attitudes toward artificial intelligence (Beta = 0.212) was significantly associated with a higher social media dependency.

**Conclusion:**

The LSMDS represents a psychometrically sound, culturally relevant instrument for assessing social media dependency among Lebanese university students. Its three-factor structure captures distinct dimensions of social media dependency, providing researchers and practitioners with a valuable tool to identify and understand social media dependency patterns in the Lebanese context.

## Introduction

Over the past decade, the rapid expansion of social media into daily life has reshaped the way individuals communicate, access information, and form social relationships [1]. Social media has grown in importance and prevalence, with an estimated number of users in 2023 at 4.9 billion worldwide [2], a figure projected to approach 6 billion by 2027 [3]. Common social media platforms include Facebook, Instagram, TikTok, X (formerly Twitter), LinkedIn, Snapchat, and WhatsApp. They can be used for both personal and professional reasons [4]. While these platforms offer numerous benefits, including enhanced connectivity and access to information, growing evidence suggests that excessive or maladaptive use may lead to negative psychological, academic, and social outcomes, including anxiety, depression, sleep disturbance, and reduced life satisfaction [5–10]. Beyond psychological outcomes, problematic social media use has also been linked to reduced physical activity levels through nomophobia and increased avoidance of exercise, highlighting the broad functional impairment associated with dependency [11]. Social media has become deeply embedded in everyday life, especially among younger populations, influencing how individuals communicate, socialize, and perceive themselves and others [12].

In fact, the constant availability of content and the pressure for social comparison may lead to compulsive use and dependency-like behaviors [13,14]. These effects are not limited to adolescents; young adults, especially university students, are also increasingly affected as social media becomes a central component of academic, professional, and social life [12,15,16].

The high prevalence of social media use among university students has been associated with lower academic performance, impaired concentration, and heightened psychological distress [17–19]. Furthermore, studies during the COVID-19 pandemic have shown that social media served both as a coping tool and a source of distress, particularly through exposure to misinformation and emotionally charged content [20,21]. These findings indicate the need to clarify and quantify problematic patterns of social media engagement among young adults, particularly university students.

To address this need, various scales have been developed to assess problematic social media use [22–25]. In the Arab region specifically, several validated Arabic-language instruments exist, including the Arabic Social Media Addiction Scale (SMAS) [26] and adaptations of international tools such as the Bergen Social Media Addiction Scale [27]. However, most Western-developed tools have been validated primarily in Western populations, limiting their generalizability across cultural settings. Cultural norms, language, and media use patterns vary widely and may influence how individuals experience and report social media dependency [28]. While Arabic-language scales such as the SMAS [26] represent important advances, they were developed and validated in different Arab contexts. Lebanon presents a particularly distinct case requiring culturally adapted assessment tools.

As of January 2025, Lebanon demonstrates remarkably high digital connectivity with 5.34 million internet users (91.6% penetration rate) and 4.02 million social media users (68.9% of the total population) [29], positioning Lebanon among the highest in the Arab region for internet and social media penetration, with particularly intensive usage among university-aged populations. The Lebanese context is characterized by unique sociocultural factors, including multilingualism (Arabic, French, and English), collectivist social structures that prioritize family and peer connections, and recent socioeconomic crises that have intensified reliance on digital communication as traditional infrastructure deteriorated [30,31].

Given the distinctive Lebanese context, which includes a trilingual media environment, collectivist values amplifying social comparison pressures, and the intersection of economic crisis with digital dependency, existing Arabic scales may not fully capture the culturally-specific manifestations of social media dependency in this population. Therefore, the current study aims to address this gap by developing and validating a Lebanese social media dependency scale (LSMDS) and exploring its correlates. The LSMDS was designed to address several specific needs: (1) capturing the unique multilingual and multicultural characteristics of Lebanese social media use, (2) integrating multiple theoretical frameworks (smartphone addiction, fear of missing out, and behavioral addiction criteria) into a single culturally-relevant instrument, (3) providing a tool specifically validated with university students in Lebanon who represent a distinct population experiencing unique stressors including economic crisis, political instability, and educational disruptions, and (4) facilitating direct comparisons within Lebanese research contexts.

## Methods

### Study design and participants

This quantitative cross-sectional study was conducted between March 3^rd^ and April 28^th^, 2025 among university students enrolled in multiple universities across Lebanon, including Lebanese International University (private, Beqaa and Beirut campuses), Lebanese University (public, multiple governorates), and other institutions distributed across Lebanon’s governorates. Recruitment targeted institutions spanning all eight governorates; however, geographic representation was uneven, with Baalbek-Hermel (5.1%) and Akkar (3.1%) being underrepresented relative to their population proportions, as acknowledged in the limitations. The target population included undergraduate and graduate students who were active social media users (defined as those reporting daily use). The survey was created on Google Forms and shared with participants through various methods, including direct email invitations, coordination with faculty members, outreach via student WhatsApp groups, and promotion across social media platforms such as WhatsApp, LinkedIn, Facebook, and Instagram. Participation was voluntary, and participants provided written informed consent digitally by ticking a consent checkbox on the first page of the online questionnaire before proceeding to the survey. To be eligible, participants had to be 18 or older, currently enrolled in a university in Lebanon, and report at least daily use of social media.

### Sample size calculation

The sample size was calculated following guidelines for scale development and validation research, recommending recruitment of 5–10 individuals for each scale item on the scale, with a minimum of 100–200 participants overall [32].

Given an initial number of 55 items, a minimum of 55 × 5 = 275 participants was needed. As the final item count was unknown at the time of study design, sample size justification was based solely on the initial 55-item pool. Adding a 10% buffer for potential missing data or exclusions resulted in a required minimum sample of 303 participants.

### Ethical considerations

The study protocol received approval from the Lebanese International University School of Pharmacy Ethics and Research Committee (2025ERC-012-LIUSOP). Written informed consent was obtained digitally from each participant via a mandatory consent checkbox on the first page of the online questionnaire. All procedures followed the ethical principles outlined in the Declaration of Helsinki. Participation was anonymous, and no identifying information was collected.

### Measurement tools

The questionnaire used for data collection was originally developed in English. The linguistic and cultural adaptation followed a six-stage process. (1) Forward translation: two bilingual specialists; one with expertise in English and one in special education, independently translated the English items into Lebanese Arabic; both were native Arabic speakers. (2) Reconciliation: discrepancies between the two Arabic drafts were adjudicated through consensus discussion. (3) Back-translation: the reconciled Arabic version was independently back-translated into English by a third bilingual expert blind to the original items. (4) Comparison and refinement: the back-translated version was compared with the original to identify semantic losses; items with meaningful discrepancies were re-worded in Arabic. (5) Cultural review: two education professionals and one social scientist with expertise in Lebanese university culture reviewed all items for cultural appropriateness, ensuring platform references matched local social media usage patterns and digital terminology was familiar to Lebanese students. (6) Pilot study: ten Lebanese university students completed the questionnaire and provided verbal feedback on clarity and cultural fit; minor wording adjustments were made based on this feedback before finalization.

Before implementation, a pilot study involving 10 participants evaluated the questionnaire’s clarity, readability, and logical flow. Based on feedback from this preliminary testing, minor modifications were made to the Arabic version to enhance wording and improve structural organization; the finalized questionnaire required approximately 7–10 minutes to complete.

The survey consisted of two sections (S1 Appendix). The first part collected information on sociodemographic and lifestyle characteristics, such as age, gender, marital status, nationality, area of residence (across Lebanon’s eight governorates), employment status, household income, smoking, alcohol use, health coverage, and academic major and year. It also included questions related to digital media use and habits, where students reported average daily smartphone use, primary social media platforms used, number of active accounts, and behaviors (e.g., checking social media during lectures, bedtime phone use, and perception of academic impact).

The second section comprised the following scales:

#### Smartphone Addiction Inventory (SPAI).

This 26-item tool assesses compulsive smartphone use across four domains: compulsive behavior, functional impairment, withdrawal, and tolerance. Responses are rated on a 4-point Likert scale (1 = strongly disagree to 4 = strongly agree) [22,33].

#### Online Fear of Missing Out Inventory (ON-FoMO).

This scale includes 20 items organized into four distinct dimensions: (1) Need to Belong, (2) Need for Popularity, (3) Anxiety, and (4) Addiction. The items are rated on a 4-point Likert scale from 1 (has nothing to do with me) to 4 (has a lot to do with me). Higher scores indicate greater fear of missing out online [23,34].

#### Social Media Disorder Scale (SMD).

A 9-item scale based on DSM-5 criteria for behavioral addictions, answered in binary format (Yes/No), assessing compulsive social media behaviors over the past year [24].

#### Attitudes towards Artificial Intelligence Scale (ATTARI-12).

This 12-item scale is designed to measure general attitudes toward artificial intelligence across cognitive, affective, and behavioral facets. It employs a Likert-type response format, allowing participants to indicate their level of agreement with statements about artificial intelligence. The scale produces a unified attitude score that reflects overall disposition toward AI technology [25].

### Statistical analysis

SPSS version 27 was used for data analysis. For descriptive analysis, quantitative measures were expressed as means and standard deviations (SD), whereas categorical variables were expressed as absolute frequencies and percentages.

The LSMDS was developed using a multi-stage psychometric process. An initial pool of 55 items was generated from three existing social media instruments: SMD, ON-FoMO, and SPAI. Item selection was guided by both content validity and statistical criteria. Prior to any statistical screening, two specialists in psychology and public health reviewed all 55 items for relevance to social media dependency in the Lebanese university context, cultural appropriateness, and conceptual clarity; items judged as culturally incongruent, excessively redundant, or poorly aligned with the target construct were flagged for removal or modification at this stage. Internal consistency was first assessed, and items with low corrected item–total correlations (<0.30) were removed. An exploratory factor analysis with oblimin rotation was then conducted, given the expected correlations between items. The number of factors to retain was determined by three converging criteria: (1) eigenvalue greater than 1 (Kaiser criterion), (2) visual inspection of the scree plot, and (3) parallel analysis based on common factor extraction. Parallel analysis supported a three-factor solution, consistent with the scree plot and theoretical interpretability (S2 Appendix). Items were removed if they had low factor loadings (<0.40), cross-loaded on multiple factors, or showed conceptual redundancy. Reliability was reevaluated after each step for both the overall scale and the individual factors. This iterative process resulted in a final set of 27 items with excellent internal consistency.

The reliability of the scale was assessed using both Cronbach’s alpha and McDonald’s ω, providing estimates of internal consistency for the overall scale and its subscales. Using AMOS software, a confirmatory factor analysis (CFA) was conducted on a random 50% subsample to evaluate the proposed dimensionality of the model. Model fit was assessed using the following indices, with bootstrapping used: the chi-square to degrees of freedom ratio (χ²/df), Root Mean Square Error of Approximation (RMSEA), Comparative Fit Index (CFI), and Tucker–Lewis Index (TLI). A χ²/df ratio between 2 and 5 indicates an acceptable fit. RMSEA values below 0.05 suggest a close fit, while values below 0.11 indicate an acceptable fit. For the CFI and TLI, values ≥ 0.90 reflect a well-fitting model.

Convergent validity of the LSMDS was then evaluated through Pearson correlations between the LSMDS subscale scores and social media scales used in the study.

Bivariate analysis was then used for comparisons between three or more groups using the ANOVA test and the independent samples t-tests for comparisons between two groups. Additionally, Pearson correlation coefficients were used to evaluate relationships between continuous variables. After that, a linear regression analysis using the Enter method was performed with the LSMDS as the dependent variable. The multivariable model included sociodemographic variables (gender, marital status, employment status, age) and the ATTARI-12 scale, all selected based on their theoretical relevance to social media dependency as identified in prior literature. The SPAI, ON-FoMO, and SMD scales were excluded to avoid multicollinearity arising from their high correlations with the LSMDS (r = 0.621–0.888). Multicollinearity was assessed using Variance Inflation Factors (VIF); all values were below 5 (range: 1.02–3.87), indicating no problematic multicollinearity among predictors. Model fit was evaluated using R² and adjusted R².. Statistical significance was set at p < 0.05.

## Results

### Sample description

A total of 511 university students participated in this study and were included in the final analysis.

Table 1 presents participants’ sociodemographic characteristics. Most participants were Lebanese (83.0%), female (70.6%), single (97.1%), and unemployed (75.3%), and 49.0% reported intermediate to low monthly income. Only 34.6% of participants resided in the Beirut and Mount Lebanon regions. The mean age was 21.04 ± 3.52 years.

**Table 1:** Sociodemographic characteristics of the participants (N = 511).

	N (%)
**Gender**	
Male	150 (29.4%)
Female	361 (70.6%)
**Marital status**	
Single/ widowed/ divorced	496 (97.1%)
Married	15 (2.9%)
**Nationality**	
Lebanese	424 (83.0%)
Non-Lebanese	87 (17.0%)
**Academic major**	
Pharmacy	38 (7.4%)
Medicine	4 (0.8%)
Engineering	73 (14.3%)
Education	44 (8.6%)
Business	99 (19.4%)
Art & Sciences	212 (41.5%)
Others	41 (8.0%)
**Year of study**	
First year undergraduate	198 (38.7%)
Second year undergraduate	115 (22.5%)
Third year undergraduate	116 (22.7%)
Fourth year undergraduate	39 (7.6%)
Fifth year undergraduate	6 (1.2%)
Master student	30 (5.9%)
Other	7 (1.4%)
**Area of residence**	
Beirut	112 (21.9%)
Mount Lebanon	65 (12.7%)
North	71 (13.9%)
Akkar	16 (3.1%)
South	100 (19.6%)
Nabatieh	21 (4.1%)
Beqaa	100 (19.6%)
Baalbek	26 (5.1%)
**Household monthly income**	
Don’t know/ I prefer not to answer	248 (48.5%)
Low (<200$)	146 (28.6%)
Intermediate (500–2000$)	104 (20.4%)
High (>2000$)	13 (2.5%)
**Occupation**	
Unemployed	385 (75.3%)
Employed	126 (24.7%)
**Health coverage**	
Yes	274 (53.6%)
No	237 (46.4%)
**Smoking status**	
Yes	62 (12.1%)
No	449 (87.9%)
**Alcohol status**	
Yes	47 (9.2%)
No	464 (90.8%)
	**Mean ± SD**
**Age**	21.04 ± 3.52

### Construct validity

Table 2 presents the construct validity and reliability of LSMDS. This scale was developed by selecting items from three existing scales. Using exploratory factor analysis with oblimin rotation, we identified a clear factor structure. The number of factors to retain was determined using multiple criteria, including eigenvalues (>1), scree plot inspection, and parallel analysis based on common factor extraction. Parallel analysis supported a three-factor solution (supplementary file).

**Table 2:** Factor analysis of the Lebanese Social Media Dependency Scale (LSMDS).

Oblimin rotated matrix				
Factor		Factor 1	Factor 2	Factor 3
I feel distressed or down once I cease using smartphone for a certain period of time	SPAI_10	0.791		
I use smartphone for a longer period of time and spend more money than I had intended	SPAI_6	0.757		
I find that I have been hooking on smartphone longer and longer	SPAI_3	0.752		
I feel uneasy once I stop using the smartphone for a certain period of time	SPAI_2	0.727		
I feel restless and irritable when the smartphone is unavailable	SPAI_4	0.719		
I feel like I am missing something after stopping smartphone for a certain period of time	SPAI_16	0.715		
I fail to control the impulse to use smartphone	SPAI_11	0.711		
I feel very vigorous upon smartphone use regardless of the fatigue experienced	SPAI_5	0.708		
I feel the urge to use my smartphone again right after I stop using it	SPAI_19	0.702		
My life would be joyless if there had not been a smartphone	SPAI_20	0.583		
Surfing the smartphone has exercised negative effects on my physical health	SPAI_21	0.551		
I need people to like or comment on my posts	NeedforPopularity3		0.830	
I get annoyed when my posts do not get many likes and/or comments	NeedforPopularity1		0.804	
I would like to have more likes and/or comments on my posts	NeedforPopularity5		0.775	
I get annoyed when my friends do not tag me in posts	NeedtoBelong2		0.686	
I feel distant from people when I see them happy in posts	NeedtoBelong5		0.613	
When I see on a social network that a friend is somewhere where I wanted to go too, I feel bad	NeedtoBelong1		0.612	
I only post photos or videos that I know my friends will like	NeedforPopularity2		0.602	
I often feel sad seeing on social networks that people are happier than I am	NeedtoBelong4		0.585	
I think a lot about social networks when I do not have access to them	Anxiety3			0.738
I usually feel irritated by staying disconnected from social networks too long	Anxiety5			0.730
I get restless when I cannot access social networks	Anxiety4			0.717
If I do not have access to social networks, I think of ways to get connected	Anxiety2			0.696
I get anxious when my cell phone does not have internet signal	Anxiety1			0.688
I often feel bad when I cannot use social media	SMD_3			0.594
I regularly find that I can’t think of anything else but the moment I will be able to use social media again	SMD_1			0.550
I regularly feel dissatisfied because I want to spend more time on social media	SMD_2			0.530
**Percentage variance explained = 52.91%**				
**Cronbach alpha = 0.931**		0.913	0.866	0.847
**McDonald’s ω = 0.933**		0.914	0.860	0.881
**Kaiser-Meyer-Olkin (KMO)= 0.939**				
**Bartlett’s test of sphericity p < 0.001**				

**Factor 1 (11 items): Problematic Smartphone Use.**

**Factor 2 (8 items): Social Media Validation Seeking.**

**Factor 3 (8 items): Social Media Withdrawal Symptom.**

Of the initial 55 items from all three scales, 27 items were retained based on their factor loadings and relevance. The factor analysis revealed a three-factor solution explaining 52.91% of the total variance (KMO = 0.939; Bartlett’s test of sphericity p < 0.001). The first factor, Problematic Smartphone Use, consisted of 11 items, the second factor, Social Media Validation Seeking, included 8 items, and the third factor, Social Media Withdrawal Symptom, comprised 8 items. The overall scale demonstrated excellent reliability, with Cronbach’s alpha coefficients of 0.931 and McDonald’s ω of 0.933.

### Confirmatory factor analysis

The model demonstrated a marginally acceptable fit, with a χ²/df of 2.544, indicating reasonable adequacy. The RMSEA was 0.079 (95% CI: 0.072–0.085), which falls within the range of an acceptable fit. The fit indices were below the commonly recommended threshold of 0.90, with CFI = 0.857 and TLI = 0.843, indicating moderate rather than excellent model fit (Table 3).

**Table 3:** Confirmatory factor analysis of the Lebanese Social Media Dependency Scale (N = 251).

	χ2	df	x2/df	RMSEA (95% CI)	CFI	TLI
Lebanese Social Media Dependency Scale (LSMDS)	816.917	321	2.544	0.079 (0.072, 0.085)	0.857	0.843

### Convergent validity

Table 4 presents the correlation coefficients between the LSMDS total score and its three subscales, as well as their associations with the social media scales used in the current study. The LSMDS scale was strongly intercorrelated with the three subscales. A positive correlation was found with factor 1 (r = 0.883, p < 0.001), factor 2 (r = 0.758, p < 0.001), and factor 3 (r = 0.842, p < 0.001).

**Table 4:** Correlation between the Lebanese Social Media Dependency Scale (LSMDS) and the social media scales used in the study.

	LSMDS scale	Factor 1	Factor 2	Factor 3
**Factor 1**	0.883	**–**		
*p-value*	**<0.001**	**–**		
**Factor 2**	0.758	0.439	–	
*p-value*	**<0.001**	**<0.001**	**–**	
**Factor 3**	0.842	0.637	0.540	**–**
*p-value*	**<0.001**	**<0.001**	**<0.001**	**–**
**Attitudes towards artificial intelligence Scale (ATTARI-12)**	0.106	0.069	0.066	0.149
*p-value*	**0.016**	0.121	0.137	**<0.001**
**The Smartphone Addiction Inventory (SPAI)**	0.863	0.947	0.472	0.624
*p-value*	**<0.001**	**<0.001**	**<0.001**	**<0.001**
**Online Fear of Missing Out Inventory (ON-FoMO)**	0.888	0.603	0.888	0.804
*p-value*	**<0.001**	**<0.001**	**<0.001**	**<0.001**
**Social Media Disorder Scale (SMD)**	0.621	0.533	0.413	0.613
*p-value*	**<0.001**	**<0.001**	**<0.001**	**<0.001**

Factor 1 (11 items): Problematic Smartphone Use.

Factor 2 (8 items): Social Media Validation Seeking.

Factor 3 (8 items): Social Media Withdrawal Symptom.

**Values marked in bold are significant.**

Significant positive correlations were found between the LSMDS and social media-related scales, supporting LSMDS convergent validity. The SPAI showed a very strong correlation with the LSMDS (r = 0.863, p < 0.001). The ON-FoMO also correlated strongly with the total LSMDS score (r = 0.888, p < 0.001). Moderate correlations were observed between the LSMDS and the SMD (r = 0.621, p < 0.001), and a weak correlation was found between the LMSDS and ATTARI-12 (r = 0.106, p = 0.016).

### Bivariate analysis

The bivariate analysis taking the LSMDS as the dependent variable is presented in Table 5. Single participants exhibited a significantly higher mean LSMDS score compared to married participants (47.50 vs. 39.33, p = 0.037). Other variables, such as gender, nationality, area of residence, monthly income, occupation, and age, did not show any significant association with the LSMDS scale (p > 0.05 for all).

**Table 5:** Bivariate analysis taking the Lebanese Social Media Dependency Scale (LSMDS) as the dependent variable.

	LSMDS scale	p-value
	Mean ± SD	
**Gender**		
Male	46.36 ± 15.28	0.380
Female	47.63 ± 14.83	
**Marital status**		
Single/ widowed/ divorced	47.50 ± 14.87	**0.037**
Married	39.33 ± 16.31	
**Nationality**		
Lebanese	47.14 ± 14.70	0.693
Non-Lebanese	47.83 ± 16.21	
**Area of residence**		
Beirut	46.55 ± 15.40	0.803
Mount Lebanon	46.43 ± 15.64	
North	48.47 ± 13.78	
Akkar	47.06 ± 16.14	
South	45.87 ± 15.62	
Nabatieh	47.61 ± 14.64	
Beqaa	49.33 ± 14.42	
Baalback	46.30 ± 14.18	
**Household monthly income**		
Don’t know/ I prefer not to answer	46.89 ± 14.81	0.385
Low (<200$)	48.97 ± 15.37	
Intermediate (500–2000$)	45.99 ± 14.81	
High (>2000$)	45.23 ± 14.21	
**Occupation**		
Unemployed	47.84 ± 15.04	0.121
Employed	45.46 ± 14.63	
	**Correlation coefficient**	
**Age**	−0.067	0.130

**Values marked in bold are significant.**

### Multivariable analysis

A linear regression model was performed with LSMDS as the dependent variable. Higher ATTARI scores (Beta = 0.212) were significantly associated with higher LSMDS scores. The other variables did not reach statistical significance (Table 6).

**Table 6:** Multivariable linear regression analysis taking the Lebanese Social Media Dependency Scale (LSMDS) as the dependent variable.

Correlates of the LSMDS scale					
	Unstandardized Beta	Standardized Beta	p-value	Confidence interval	
				Lower Bound	Upper Bound
Marital status (Married vs single*)	−7.182	−0.081	0.117	−16.157	1.793
Employment status	−1.824	−0.053	0.259	−4.993	1.346
Gender (Female vs male*)	1.564	0.048	0.289	−1.331	4.459
Age	−0.027	−0.006	0.906	−0.474	0.421
**ATTARI scale**	**0.212**	**0.111**	**0.013**	**0.046**	**0.378**

*Reference group.

## Discussion

This study developed and validated the Lebanese Social Media Dependency Scale (LSMDS), a culturally adapted multidimensional instrument for assessing social media dependency among Lebanese university students. The final 27-item scale comprises three factors—Problematic Smartphone Use, Social Media Validation Seeking, and Social Media Withdrawal Symptoms—explaining 52.91% of the total variance. The LSMDS demonstrated excellent internal consistency (Cronbach’s α = 0.931; McDonald’s ω = 0.933) and strong convergent validity with established social media dependency measures. Confirmatory factor analysis indicated acceptable model fit, though some fit indices fell slightly below optimal thresholds. The LSMDS addresses a critical gap in the assessment of social media dependency within the Lebanese context, while accounting for how distinctive sociocultural characteristics, including multilingualism, collectivist values, and crisis-related factors, shape digital media engagement in this population. An important conceptual clarification is warranted regarding the scale’s name and its first factor. The LSMDS measures social media dependency as a multidimensional construct that includes; but is not reducible to, problematic smartphone use. In the Lebanese university context, social media access is almost exclusively mediated through smartphones; accordingly, Factor 1 captures the compulsive, device-mediated mode through which social media dependency is behaviorally enacted, rather than generic device overuse disconnected from social media content. The three factors together constitute an integrated model: compulsive smartphone-based social media access (Factor 1), the motivational-affective drive toward online validation (Factor 2), and the neurobiological withdrawal-like consequences of disrupted access (Factor 3). While the item content draws on internationally recognized indicators of social media dependency, the cultural contribution of the LSMDS lies in validating this factor structure within the Lebanese university context and demonstrating its reliability and interpretability for Arabic-speaking Lebanese students, rather than in generating wholly novel items.

### Construct validity

The three-factor structure of the LSMDS is consistent with other Arabic-language social media dependency instruments. The Arabic Social Media Addiction Scale (SMAS) similarly demonstrated a three-factor model in undergraduate students [26,35], supporting the validity of this dimensional approach in Arab populations.

The first factor, Problematic Smartphone Use (11 items), captured behaviors and experiences such as distress when ceasing smartphone use, inability to control usage impulses, restlessness or irritability without smartphone access, and physical health impacts. This dimension was identified as a fundamental aspect of social media dependency, influencing both mental health and daily functioning [36]. The emotional dysregulation dimension of this factor is consistent with longitudinal evidence that escape from negative emotions and mood enhancement motives mediate the relationship between psychological distress and problematic smartphone use [37]. Theoretically, this factor is grounded in the behavioral addiction model, which conceptualizes compulsive technology use as sharing core features with substance-related addictions; including salience, tolerance, mood modification, and loss of control, and in impulse control theories, which explain the inability to resist urges despite awareness of negative consequences.

The second factor, Social Media Validation Seeking (8 items), reflected the need for social approval through likes, comments, and attention to posts. Similar research showed that young adults experience alleviation of inadequacy feelings through online validation. This aligns with evidence that social media dependency is associated with lower self-esteem and heightened loneliness, with individuals using platforms to seek the social approval and sense of belonging that is deficient in their offline lives [38]. However, this validation creates a dopamine-driven feedback loop that can worsen pre-existing mental health conditions [39]. This mechanism operates similarly to gaming devices, establishing dependency through uncertain reward patterns [6]. Theoretically, this factor is grounded in social comparison theory, which posits that individuals evaluate their own worth relative to others, and in self-determination theory, particularly the basic psychological needs for competence (seeking validation of one’s abilities and social standing) and relatedness (the need to feel connected and accepted by peers). When these needs are unmet in offline contexts, social media platforms become a substitute arena for their fulfillment, driving compulsive validation-seeking behavior.

The third factor, Social Media Withdrawal Symptoms (8 items), assessed anxiety and restlessness associated with disconnection from social networks. A Romanian study identified salience and withdrawal as the most central symptoms of social media dependency [40]. Further research demonstrated that withdrawal symptoms mediate the relationship between social media use and psychological distress among adolescents and young adults [41]. Theoretically, this factor is grounded in the neurobiological underpinnings of behavioral addictions, specifically the role of dopaminergic reward pathways in reinforcing platform engagement. Repeated activation of these pathways leads to neuroadaptation, manifesting as tolerance (requiring increasing use to achieve the same effect) and withdrawal (negative affective and physiological states upon cessation), mirroring the mechanisms observed in substance-use disorders and providing a biological basis for the compulsive disconnection anxiety captured by these items. Moreover, the high rates of anxiety and depression co-occurring with social media dependency in non-Western samples support the conceptualization of withdrawal symptoms as not merely behavioral but embedded within broader patterns of psychological distress [42].

The LSMDS showed excellent overall reliability (Cronbach’s alpha of 0.931; McDonald’s ω = 0.933) with strong internal consistency for each subscale (alpha range: 0.847–0.913). Sampling adequacy was confirmed through a Kaiser-Meyer-Olkin measure of 0.939 and a highly significant Bartlett’s test of sphericity (p < 0.001). These findings provide initial support that the LSMDS is psychometrically promising, capturing distinct dimensions of social media dependency in the Lebanese context. These psychometric properties are consistent with recent Arabic-language instruments, including the Problematic Use of Social Networks (PUSN) scale Arabic version, which showed excellent reliability and confirmed factor validity among Lebanese adolescents [43], and the Arabic Social Media Addiction Scale (SMAS), which also exhibited a three-factor structure with strong internal consistency and construct validity [26].

### Convergent validity

The LSMDS total score demonstrated strong positive correlations with its three subscales and established social media-related measures. The strongest correlation was observed with the ON-FoMO, followed by the SPAI, and a moderate correlation with the SMD. These findings align with research indicating substantial overlap between smartphone dependency, fear of missing out, and social media dependency [43]. The particularly robust correlations with ON-FoMO and SPAI suggest that fear of missing out and problematic smartphone use are central psychological mechanisms underlying social media dependency in university students in Lebanon. The positive correlation with SMD provides additional evidence for the scale’s validity, despite differences in measurement approach (binary vs. Likert scale), indicating that the LSMDS captures problematic social media behaviors consistent with behavioral addiction criteria. It should be acknowledged, however, that because the LSMDS items were derived from the SPAI, ON-FoMO, and SMD, the strong correlations observed with these parent measures partly reflect shared item origins and conceptual overlap rather than constituting fully independent convergent validity evidence. Future validation studies should assess the LSMDS against measures with which it shares no item ancestry to provide more independent evidence of validity.

These findings also align with validation studies of social media addiction scales across various Arab cultural contexts. The Social Media Addiction Scale-Student Form (SMAS-SF), validated in Saudi Arabia, similarly demonstrated strong convergent validity with related psychological and behavioral constructs [44], supporting the LSMDS as a culturally appropriate assessment tool for Arab populations.

Notably, only a weak correlation was found between the LSMDS and the ATTARI-12, suggesting that attitudes toward AI technology and social media dependency are largely independent constructs at the bivariate level. However, this relationship became statistically significant in multivariable analysis, indicating that the association between these variables may be influenced by other demographic or behavioral factors. This finding is consistent with evidence that algorithmic recommendation-driven engagement is a distinct motivational driver of social media dependency, separate from social or entertainment motives, suggesting that users who embrace AI-curated content may be particularly vulnerable to dependency-sustaining feedback loops [45].

### Factors affecting the LSMDS scale

Bivariate analysis revealed that single participants have a significantly higher mean LSMDS score compared to married participants. This finding aligns with studies in the Lebanese context showing that unmarried or single individuals exhibit higher levels of problematic smartphone and social media use [46,47]. This association may reflect the role of loneliness as a driver of social media engagement, as research indicates that feelings of loneliness are associated with increased social media dependency [38]. It may also reflect greater social needs, fewer familial responsibilities, and potentially more discretionary time available for social media engagement among single individuals, who may also rely more heavily on digital platforms to maintain social connections and fulfill belonging needs typically met through family relationships.

However, marital status did not retain statistical significance as a predictor of LSMDS scores in multivariable regression analysis when controlling for sociodemographic variables. This finding suggests that the bivariate relationship may be confounded by unmeasured variables or that the effect size is too small to detect with multiple predictors included. These findings should also be interpreted in light of the sample’s demographic composition. The overrepresentation of female participants (70.6%) means that findings may not generalize equally to male university students; although gender did not emerge as a significant predictor in multivariable analysis, this null finding may partly reflect reduced statistical power to detect gender differences given the uneven distribution. This demographic pattern is, however, consistent with survey-based research in Lebanon and the broader Arab region, where female students tend to participate at higher rates in online studies. Similarly, the near-universal proportion of single participants (97.1%) substantially limits the generalizability of the marital status finding; with only 15 married participants, the observed difference in LSMDS scores between single and married individuals should be treated as preliminary and interpreted with considerable caution. Future studies should employ stratified recruitment to ensure adequate representation of male and married students before drawing firm conclusions about the moderating roles of gender and marital status on social media dependency.

The only significant predictor in the multivariable model was attitudes toward AI, with higher ATTARI-12 scores significantly associated with higher LSMDS scores. Given the weak bivariate correlation, this finding was unexpected and represents a novel contribution to the literature, suggesting that the relationship between AI attitudes and social media dependency emerges only when accounting for other factors.

This finding aligns with recent research [48] that investigated associations between AI attitudes and problematic social media use in 956 German social media users. The study found that positive AI attitudes were significantly linked to more severe problematic social media, with this association being mediated by time spent on social media. Individuals with more positive attitudes toward AI scored higher on the Social Networking Sites-Addiction Test. This effect was more pronounced in male participants. Mediation analysis showed that favorable attitudes toward AI predicted increased time spent on social media, which, in turn, predicted problematic social media use patterns [48].

Several mechanisms may explain this relationship. First, individuals with favorable attitudes toward AI may be more receptive to digital technologies, leading to more intensive social media use [49]. Second, those who view AI favorably may be early adopters of AI-enhanced social media features (such as personalized content algorithms, chatbots, and recommendation systems), which could increase platform engagement and dependency. Third, favorable attitudes toward AI might reflect a broader comfort with algorithmic curation of content, a core feature of modern social media platforms designed to maximize user engagement and time spent on platforms. Fourth, individuals interested in AI may spend more time on technology-focused social media communities and discussions, inadvertently increasing their overall social media use [49,50].

### Limitations

Several limitations should be acknowledged when interpreting the findings of this study. First, the cross-sectional design prevents establishing causal relationships and assessing the temporal stability of the LSMDS. Furthermore, data collection relied exclusively on self-report measures, introducing potential response, social desirability, or recall bias. Participants may have underreported or overreported their social media behaviors due to the stigma associated with dependency-like behaviors or the lack of accurate self-awareness regarding their usage patterns. Related to sampling concerns, online recruitment may have created selection bias toward active social media users, potentially excluding those with minimal engagement or those who have already reduced their social media use due to prior negative experiences. Additionally, despite targeting all eight governorates, geographic representation was uneven: Baalbek-Hermel (n = 26, 5.1%) and Akkar (n = 16, 3.1%) were notably underrepresented relative to their population proportions, likely due to the online and university-network-based recruitment method, which may have favoured institutions geographically closer to major urban centres. This limits the generalizability of findings to students from more remote or rural regions of Lebanon. The sample was predominantly female (70.6%) and overwhelmingly single (97.1%),

which substantially restricts generalizability to male students and married individuals; the very small married subsample (n = 15) means that the significant bivariate finding regarding marital status should be interpreted with particular caution. Future studies should employ stratified or quota sampling to achieve balanced representation across gender and marital status categories. The voluntary nature of participation may have attracted individuals particularly interested in or concerned about their social media use. Moreover, the factor structure requires confirmation through independent sample validation. While confirmatory factor analysis was conducted on a random 50% subsample, which showed acceptable fit indices, independent replication would strengthen confidence in the three-factor structure. Also, the absence of test-retest reliability data means that the temporal stability of LSMDS scores has not been established; future studies should include a test-retest component to demonstrate score consistency over time. The lack of discriminant validity evidence means it cannot yet be confirmed that the LSMDS is sufficiently distinct from related constructs such as general internet addiction or depression, and criterion validity against clinical measures of social media disorder was not assessed. Future research should examine whether LSMDS scores predict clinically relevant outcomes such as academic impairment, psychological distress, or functional difficulties in daily life.

### Strengths and clinical implications

This study presents several notable strengths that enhance the value of the LSMDS as a clinical and research tool. The robust sample size of 511 university students exceeds recommended guidelines for scale validation, ensuring statistical power and generalizability within the Lebanese context. The systematic methodology, including rigorous translation procedures, pilot testing, and comprehensive psychometric evaluation, strengthens confidence in the scale’s reliability and validity. The LSMDS demonstrated excellent internal consistency and strong reliability for individual factors, indicating that the scale produces consistent and dependable measurements. The three-factor structure provides clinicians and mental health practitioners with a framework for understanding different manifestations of social media dependency—distinguishing between problematic smartphone behaviors, validation-seeking patterns, and withdrawal symptoms. This specificity enables targeted interventions tailored to individual symptom profiles rather than treating social media dependency as a monolithic condition. From a clinical perspective, the LSMDS can serve as a screening tool in university counseling centers, healthcare settings, and mental health services to identify students at risk for problematic social media use before it escalates to clinically significant impairment. The scale’s cultural adaptation makes it particularly valuable for practitioners working with Lebanese and potentially other Arab populations, addressing a substantial gap in regionally appropriate assessment instruments. Furthermore, the identification of attitudes toward AI as a significant predictor of social media dependency, though requiring further investigation, suggests a novel avenue for prevention. As AI becomes increasingly integrated into social media platforms through personalized content algorithms, chatbots, and recommendation systems, understanding how attitudes toward these technologies relate to dependency patterns may inform psychoeducational interventions. Programs aimed at promoting digital literacy and critical awareness of AI-driven engagement strategies could help students develop healthier relationships with social media platforms.

## Conclusion

The LSMDS is a psychometrically promising instrument for assessing social media dependency among Lebanese university students, capturing three dimensions of social media dependency: Problematic Smartphone Use, Social Media Validation Seeking, and Social Media Withdrawal Symptoms. By accounting for Lebanon’s unique multilingual environment and sociocultural context, the scale addresses a critical gap in culturally adapted assessment tools for Arab populations. It should be noted, however, that because the LSMDS items were derived from the SPAI, ON-FoMO, and SMD scales, the strong correlations observed with these parent measures partly reflect shared item origins rather than fully independent convergent validity; future studies should therefore validate the LSMDS against measures with no shared item ancestry to provide more robust evidence of its distinctiveness.

The finding that positive attitudes toward artificial intelligence predict higher social media dependency represents a novel contribution warranting further investigation, particularly as AI-driven features become increasingly integrated into social media platforms. This validated instrument provides researchers and practitioners with a reliable means to identify at-risk individuals, monitor trends in social media use, and develop evidence-based interventions tailored to the Lebanese educational context.

## Supporting information

S1 Appendix The Study Questionnaire. (PDF)

S2 Appendix Results of the Parallel Analysis. (DOCX)
