# Supplementary material for: Development and validation of the Lebanese Social Media Dependency Scale (LSMDS): A cross-sectional study among university students
Source: PLoS One. 2026 Apr 28;21(4):e0344535. doi: 10.1371/journal.pone.0344535 (PMC13123986; doi:10.1371/journal.pone.0344535)
Supplement: S1 Appendix — (PDF) [file pone.0344535.s001.pdf]

Social Media Use, Fear of Missing Out, and Well-being Among University Students

استخدام وسائل التواصل الاجتماعي، والخوف من تفويت الفرص، والرفاهية بين طلاب الجامعات

Dear Student, / عزيزي الطالب(ة)،

You are invited to participate in a research study examining the relationship between smartphone use, social media habits, and well-being among university students. This survey will take approximately 10 minutes to complete. Your participation is voluntary, and your responses will remain completely confidential. The information you provide will help us better understand the impact of digital technology on student life and may contribute to developing better support services for students. There are no right or wrong answers. Please respond to all questions as honestly as possible. You may discontinue the survey at any time. By completing this survey, you consent to participate in this research study.

أنت مدعو للمشاركة في دراسة بحثية تفحص العلاقة بين استخدام الهاتف الذكي، وعادات وسائل التواصل الاجتماعي، والرفاهية بين طلاب الجامعات. سيستغرق إكمال هذا الاستطلاع حوالي 10 دقائق. مشاركتك طوعية، وستبقى إجاباتك سرية تمامًا. المعلومات التي تقدمها ستساعدنا على فهم تأثير التكنولوجيا الرقمية على حياة الطلاب بشكل أفضل وقد تساهم في تطوير خدمات دعم أفضل للطلاب. لا توجد إجابات صحيحة أو خاطئة. يرجى الإجابة على جميع الأسئلة بصدق قدر الإمكان. يمكنك التوقف عن الاستطلاع في أي وقت. بإكمالك لهذا الاستطلاع، فإنك توافق على المشاركة في هذه الدراسة البحثية.

Thank you for your valuable contribution to this research.

شكرًا لمساهمتك القيمة في هذا البحث.

The Research Team / فريق البحث

Informed consent / الموافقة المستنيرة

Please check all the boxes to proceed to the survey / يرجى تحديد جميع المربعات للمتابعة إلى الاستطلاع

☐ I have read and understood the above information / لقد قرأت وفهمت المعلومات المذكورة أعلاه

☐ I understand that my participation is voluntary / أفهم أن مشاركتي طوعية

☐ I understand that my data will be kept confidential / أفهم أن بياناتي ستبقى سرية

☐ I agree to participate in this study / أوافق على المشاركة في هذه الدراسة

Demographics Section / قسم البيانات الديموغرافية

Age in years / العمر بالسنوات _______

Gender / الجنس

- Male / ذكر
- Female / أنثى

Nationality / الجنسية

- Lebanese / لبناني
- Non-Lebanese / غير لبناني

Area of residence / منطقة السكن

- Bekaa / البقاع
- Baalbeck/Hermel / بعلبك/الهرمل
- Mount Lebanon / جبل لبنان
- Beirut / بيروت
- North / الشمال
- South / الجنوب
- Akkar / عكار
- Nabatieh / النبطية

Marital status / الحالة الاجتماعية

- Single / أعزب
- Married / متزوج
- Divorced/Widowed/Separated / مطلق/أرمل/منفصل

Type of university you attend / نوع الجامعة التي تدرس فيها

- Private / خاصة
- Public / عامة

Name of the university where you are enrolled / اسم الجامعة التي أنت مسجل فيها

- Lebanese University / الجامعة اللبنانية
- Lebanese International University / الجامعة اللبنانية الدولية
- American University of Beirut / الجامعة الأمريكية في بيروت
- Beirut Arab University / جامعة بيروت العربية
- Lebanese American University / الجامعة اللبنانية الأمريكية
- American University of Science and Technology / الجامعة الأمريكية للعلوم والتكنولوجيا

- Saint Joseph University of Beirut / جامعة القديس يوسف في بيروت
- University of Balamand / جامعة البلمند
- Rafik Hariri University / جامعة رفيق الحريري
- Modern University for Business and Science / الجامعة الحديثة للأعمال والعلوم
- Others / أخرى

School / الكلية

- Pharmacy / الصيدلة
- Medicine / الطب
- Engineering / الهندسة
- Education / التربية
- Business / الأعمال
- Arts & Sciences / الآداب والعلوم
- Nursing / التمريض
- Others / أخرى

Year of study / سنة الدراسة

- First year undergraduate / السنة الأولى جامعية
- Second year undergraduate / السنة الثانية جامعية
- Third year undergraduate / السنة الثالثة جامعية
- Fourth year undergraduate / السنة الرابعة جامعية
- Fifth year undergraduate / السنة الخامسة جامعية
- Sixth year undergraduate / السنة السادسة جامعية
- Masters student / طالب ماجستير
- Doctoral student / طالب دكتوراه
- Other (please specify) / أخرى (يرجى التحديد) ________

Current GPA (or average/20) /(20 / المعدل التراكمي الحالي (أو المعدل العام

Do you drink alcohol? / هل تشرب الكحول؟

- Yes, frequently (more than once weekly) / نعم، بشكل متكرر (أكثر من مرة أسبوعيًا)
- Yes, occasionally (once weekly or less) / نعم، أحيانًا (مرة أسبوعيًا أو أقل)
- No / لا

Do you smoke cigarettes? / هل تدخن السجائر؟

- Yes, current smoker / نعم، مدخن حالي

- Yes, previous smoker / نعم، مدخن سابق
- No / لا

Do you smoke nargileh? / هل تدخن النرجيلة؟

- Yes, current smoker / نعم، مدخن حالي
- Yes, previous smoker / نعم، مدخن سابق
- No / لا

Do you use e-cigarettes/vaping devices? / هل تستخدم السجائر الإلكترونية/أجهزة التدخين الإلكتروني؟

- Yes, currently / نعم، حاليًا
- Yes, I used to / نعم، اعتدت ذلك
- No / لا

How many individuals live in your household, including yourself? / كم عدد الأفراد الذين يعيشون في منزلك، بما في ذلك أنت؟_______

Household monthly income / الدخل الشهري للأسرة

- < 200$
- 200-499$
- 500-999$
- 1000-2000$
- > 2000$
- Don't know/I prefer not to answer / لا أعرف/أفضل عدم الإجابة

Do you have any health coverage? / هل لديك أي تغطية صحية؟

- Yes, NSSF / نعم، الصندوق الوطني للضمان الاجتماعي
- Yes, COOP / نعم، تعاونية موظفي الدولة
- Yes, army / نعم، الجيش
- Yes, private insurance / نعم، تأمين خاص
- Yes, other / نعم، أخرى
- No / لا

Current Living Situation / وضع المعيشة الحالي

- University dormitory / سكن جامعي
- Off-campus with family / خارج الحرم الجامعي مع العائلة
- Off-campus with roommates / خارج الحرم الجامعي مع زملاء السكن
- Off-campus alone / خارج الحرم الجامعي بمفردك

- Other (please specify) / أخرى (يرجى التحديد) ________

Employment Status / الوضع الوظيفي

- Not employed / غير موظف
- Part-time (≤20 hours/week) / دوام جزئي (20 ساعة/أسبوع أو أقل)
- Full-time (>20 hours/week) / دوام كامل (أكثر من 20 ساعة/أسبوع)

Digital Usage Patterns / أنماط الاستخدام الرقمي

Device Ownership (YES/NO) / ملكية الأجهزة (نعم/لا)

- Smartphone / هاتف ذكي
- Tablet / جهاز لوحي
- Laptop / كمبيوتر محمول
- Desktop computer / كمبيوتر مكتبي
- Smart watch / ساعة ذكية
- Other / أخرى

Average daily smartphone use / متوسط استخدام الهاتف الذكي اليومي

- Less than 2 hours / أقل من ساعتين
- 2-4 hours / 2-4 ساعات
- 4-6 hours / 4-6 ساعات
- 6-8 hours / 6-8 ساعات
- More than 8 hours / أكثر من 8 ساعات

Social Media Usage / استخدام وسائل التواصل الاجتماعي

a. Number of active social media accounts / عدد حسابات وسائل التواصل الاجتماعي النشطة ________

b. Most frequently used platforms (YES/NO) / المنصات الأكثر استخداماً (نعم/لا)

- Instagram / إنستغرام
- Facebook / فيسبوك
- Twitter/X / تويتر/إكس
- TikTok / تيك توك
- Snapchat / سناب شات
- LinkedIn / لينكد إن
- WhatsApp / واتساب
- YouTube / يوتيوب

- Other / أخرى

c. Primary purpose of social media use (YES/NO) / الغرض الرئيسي من استخدام وسائل التواصل الاجتماعي (نعم/لا)

- Entertainment / الترفيه
- Communication with friends/family / التواصل مع الأصدقاء/العائلة
- Academic networking / التواصل الأكاديمي
- Professional networking / التواصل المهني
- News/Information / الأخبار/المعلومات
- Content creation / إنشاء المحتوى
- Dating/Meeting new people / المواعدة/مقابلة أشخاص جدد
- Other / أخرى

Sleep Patterns / أنماط النوم

a. Average bedtime / متوسط وقت النوم _______

b. Average wake-up time / متوسط وقت الاستيقاظ _______

c. Do you use your smartphone in bed? / هل تستخدم هاتفك الذكي في السرير؟

- Never / أبدًا
- Rarely / نادرًا
- Sometimes / أحيانًا
- Often / غالبًا
- Always / دائمًا

Academic Impact / التأثير الأكاديمي

Do you use your smartphone during / هل تستخدم هاتفك الذكي خلال

a. Lectures / المحاضرات

- Never / أبدًا
- Rarely / نادرًا
- Sometimes / أحيانًا
- Often / غالبًا
- Always / دائمًا

b. Study sessions / جلسات الدراسة

- Never / أبدًا
- Rarely / نادرًا

- Sometimes / أحيانًا
- Often / غالبًا
- Always / دائمًا

c. Group work / العمل الجماعي

- Never / أبدًا
- Rarely / نادرًا
- Sometimes / أحيانًا
- Often / غالبًا
- Always / دائمًا

Do you think use of social media has affected your study timing? / هل تعتقد أن استخدام وسائل التواصل الاجتماعي قد أثر على توقيت دراستك؟

- Yes / نعم
- No / لا

Do you feel more attracted towards social media compared to study? / هل تشعر بانجذاب أكبر نحو وسائل التواصل الاجتماعي مقارنة بالدراسة؟

- Yes / نعم
- No / لا

Do you consider yourself addicted to social media? / هل تعتبر نفسك مدمنًا على وسائل التواصل الاجتماعي؟

- Yes / نعم
- No / لا

Attitudes towards artificial intelligence Scale (ATTARI-12) / مقياس المواقف تجاه الذكاء الاصطناعي (ATTARI-12)

Instruction: In the following, we are interested in your attitudes towards artificial intelligence (AI). AI can execute tasks that typically require human intelligence. It enables machines to sense, act, learn, and adapt in an autonomous, human-like way. AI may be part of a computer or online platform—but it can also be encountered in various other hardware devices such as robots.

التعليمات: فيما يلي، نحن مهتمون بمواقفك تجاه الذكاء الاصطناعي. يمكن للذكاء الاصطناعي تنفيذ مهام تتطلب عادةً ذكاءً بشريًا. يمكّن الآلات من الإحساس والتصرف والتعلم والتكيف بطريقة مستقلة شبيهة بالإنسان. قد يكون الذكاء الاصطناعي جزءًا من جهاز كمبيوتر أو منصة عبر الإنترنت - ولكن يمكن أيضًا مواجهته في مختلف الأجهزة الأخرى مثل الروبوتات.

Responses (1=*strongly disagree*, 2=disagree, 3=neutral, 4=agree, 5 = *strongly agree*) /

الاستجابات (1=لا أوافق بشدة، 2=لا أوافق، 3=محايد، 4=أوافق، 5=أوافق بشدة)

1. AI will make this world a better place / سيجعل الذكاء الاصطناعي هذا العالم مكانًا أفضل
2. I have strong negative emotions about AI / لدي مشاعر سلبية قوية تجاه الذكاء الاصطناعي

3. I want to use technologies that rely on AI / أريد استخدام التقنيات التي تعتمد على الذكاء الاصطناعي
4. AI has more disadvantages than advantages / للذكاء الاصطناعي مساوئ أكثر من الفوائد
5. I look forward to future AI developments / أتطلع إلى تطورات الذكاء الاصطناعي المستقبلية
6. AI offers solutions to many world problems / يقدم الذكاء الاصطناعي حلولاً للعديد من مشاكل العالم
7. I prefer technologies that do not feature AI / أفضل التقنيات التي لا تتضمن الذكاء الاصطناعي
8. I am afraid of AI / أنا خائف من الذكاء الاصطناعي
9. I would rather choose a technology with AI than one without it / أفضل اختيار تقنية مع الذكاء الاصطناعي على تقنية بدونه
10. AI creates problems rather than solving them / يخلق الذكاء الاصطناعي مشاكل بدلاً من حلها
11. When I think about AI, I have mostly positive feelings / عندما أفكر في الذكاء الاصطناعي، لدي مشاعر إيجابية في الغالب
12. I would rather avoid technologies that are based on AI / أفضل تجنب التقنيات المعتمدة على الذكاء الاصطناعي

The Smartphone Addiction Inventory (SPAI) / جرد إدمان الهواتف الذكية

Please answer the following items based on the response scale (1 = strongly disagree, 2 = slightly disagree, 3 = somewhat agree, 4 = strongly agree) / يرجى الإجابة على العناصر التالية بناءً على مقياس الاستجابة (1 = لا أوافق بشدة، 2 = لا أوافق قليلاً، 3 = أوافق إلى حد ما، 4 = أوافق بشدة)

1. I was told more than once that I spent too much time on smartphone / تم إخباري أكثر من مرة بأنني أقضي وقتًا طويلاً على الهاتف الذكي
2. I feel uneasy once I stop using the smartphone for a certain period of time / أشعر بعدم الارتياح بمجرد توقفي عن استخدام الهاتف الذكي لفترة معينة من الوقت
3. I find that I have been hooking on smartphone longer and longer / أجد أنني أصبحت متعلقًا بالهاتف الذكي لفترات أطول وأطول
4. I feel restless and irritable when the smartphone is unavailable / أشعر بالقلق والانزعاج عندما يكون الهاتف الذكي غير متاح
5. I feel very vigorous upon smartphone use regardless of the fatigue experienced / أشعر بنشاط كبير عند استخدام الهاتف الذكي بغض النظر عن التعب الذي أشعر به
6. I use smartphone for a longer period of time and spend more money than I had intended / أستخدم الهاتف الذكي لفترة أطول من الوقت وأنفق أكثر مما كنت أنوي
7. Although using smartphone has brought negative effects on my interpersonal relationships, the amount of time spent on the Internet remains unreduced / على الرغم من أن استخدام الهاتف الذكي قد أحدث آثارًا سلبية على علاقاتي الشخصية، إلا أن مقدار الوقت الذي أقضيه على الإنترنت لم ينخفض
8. I have slept less than 4 h more than one time due to using smartphone / لقد نمت أقل من 4 ساعات أكثر من مرة بسبب استخدام الهاتف الذكي

9. I have substantially increased the amount of time using smartphone per week in the three most recent months / لقد زدت بشكل كبير من مقدار الوقت الذي أستخدم فيه الهاتف الذكي أسبوعيًا في الأشهر الثلاثة الأخيرة
10. I feel distressed or down once I cease using smartphone for a certain period of time / أشعر بالضيق أو الانزعاج بمجرد توقفي عن استخدام الهاتف الذكي لفترة معينة من الوقت
11. I fail to control the impulse to use smartphone / أفشل في السيطرة على الاندفاع لاستخدام الهاتف الذكي
12. I find myself indulged on the smartphone at the cost of hanging out with friends / أجد نفسي منغمسًا في الهاتف الذكي على حساب قضاء الوقت مع الأصدقاء
13. I feel aches and soreness in the back or eye discomforts due to excessive smartphone use / أشعر بآلام وأوجاع في الظهر أو عدم راحة في العين بسبب الاستخدام المفرط للهاتف الذكي
14. The idea of using smartphone comes as the first thought on my mind when waking up each morning / تأتي فكرة استخدام الهاتف الذكي كأول فكرة في ذهني عند الاستيقاظ كل صباح
15. To use smartphone has had certain negative effects on my schoolwork or job performance / استخدام الهاتف الذكي كان له بعض الآثار السلبية على أدائي المدرسي أو الوظيفي
16. I feel like I am missing something after stopping smartphone for a certain period of time / أشعر وكأنني أفتقد شيئًا ما بعد التوقف عن استخدام الهاتف الذكي لفترة معينة من الوقت
17. My interaction with family members is decreased on account of smartphone use / قلّ تفاعلي مع أفراد الأسرة بسبب استخدام الهاتف الذكي
18. My recreational activities are reduced due to smartphone use / تقللت أنشطتي الترفيهية بسبب استخدام الهاتف الذكي
19. I feel the urge to use my smartphone again right after I stop using it / أشعر بالحاجة الملحة لاستخدام هاتفي الذكي مرة أخرى بعد توقفي عن استخدامه مباشرة
20. My life would be joyless if there had not been a smartphone / ستكون حياتي بلا بهجة لو لم يكن هناك هاتف ذكي
21. Surfing the smartphone has exercised negative effects on my physical health. For example, viewing smartphone when crossing the street; fumbling with one's smartphone while driving or waiting, and resulted in danger / تصفح الهاتف الذكي مارس تأثيرات سلبية على صحتي البدنية. على سبيل المثال، النظر إلى الهاتف الذكي عند عبور الشارع؛ العبث بالهاتف الذكي أثناء القيادة أو الانتظار، مما أدى إلى الخطر
22. I try to spend less time on smartphone, but the efforts were in vain / أحاول قضاء وقت أقل على الهاتف الذكي، لكن الجهود كانت عبثًا
23. I make it a habit to use smartphone and the sleep quality and total sleep time decreased / أجعل استخدام الهاتف الذكي عادة لي وانخفضت جودة النوم وإجمالي وقت النوم
24. I need to spend an increasing amount of time on smartphone to achieve the same satisfaction as before / أحتاج إلى قضاء المزيد من الوقت على الهاتف الذكي لتحقيق نفس الرضا كما كان من قبل
25. I cannot have meal without smartphone use / لا يمكنني تناول الطعام دون استخدام الهاتف الذكي
26. I feel tired during the daytime due to late-night use of smartphone / أشعر بالتعب خلال النهار بسبب استخدام الهاتف الذكي في وقت متأخر من الليل

Social Media Disorder Scale (SMD) / مقياس اضطراب وسائل التواصل الاجتماعي

Please answer the question by thinking of your experience with using social media (e.g., WhatsApp, SnapChat, Instagram, Twitter, Facebook, Google+, Pinterest, forums, weblogs) in past year. Answer the questions as honestly as possible.

يرجى الإجابة على السؤال من خلال التفكير في تجربتك مع استخدام وسائل التواصل الاجتماعي (مثل واتساب، سناب شات، إنستغرام، تويتر، فيسبوك، جوجل+، بينتيريست، المنتديات، المدونات) في العام الماضي. أجب على الأسئلة بصدق قدر الإمكان.

During the past year have you... / خلال العام الماضي هل...

1. Regularly found that you can't think of anything else but the moment you will be able to use social media again? / وجدت بانتظام أنك لا تستطيع التفكير في أي شيء آخر سوى اللحظة التي ستتمكن فيها من استخدام وسائل التواصل الاجتماعي مرة أخرى؟
2. Regularly felt dissatisfied because you want to spend more time on social media? / شعرت بعدم الرضا بانتظام لأنك تريد قضاء المزيد من الوقت على وسائل التواصل الاجتماعي؟
3. Often felt bad when you could not use social media? / شعرت غالبًا بالسوء عندما لم تتمكن من استخدام وسائل التواصل الاجتماعي؟
4. Tried to spend less time on social media, but failed? / حاولت قضاء وقت أقل على وسائل التواصل الاجتماعي، لكنك فشلت؟
5. Regularly neglected other activities (i.e. hobbies, sports, homework) because you wanted to use social media? / أهملت بانتظام أنشطة أخرى (مثل الهوايات، الرياضة، الواجبات المنزلية) لأنك أردت استخدام وسائل التواصل الاجتماعي؟
6. Regularly had arguments with others because of your social media use? / كان لديك مشاجرات منتظمة مع الآخرين بسبب استخدامك لوسائل التواصل الاجتماعي؟
7. Regularly lied to your parents or friends about the amount of time you spend on social media? / كذبت بانتظام على والديك أو أصدقائك بشأن مقدار الوقت الذي تقضيه على وسائل التواصل الاجتماعي؟
8. Often used social media to escape from negative feelings? / استخدمت غالبًا وسائل التواصل الاجتماعي للهروب من المشاعر السلبية؟
9. Had serious conflict with parents, brother, sister (friends, relationships etc.) because of your social media use? / كان لديك صراع خطير مع والديك، أخ، أخت (أصدقاء، علاقات، إلخ) بسبب استخدامك لوسائل التواصل الاجتماعي؟

Online Fear of Missing Out Inventory (ON-FoMO) / مقياس الخوف من تفويت الفرص عبر الإنترنت

Please answer the following items based on the response scale (1 = has nothing to do with me, 2 = has a little to do with me, 3 = has a moderate amount to do with me, 4 = has a lot to do with me) / يرجى الإجابة على العناصر التالية بناءً على مقياس الاستجابة (1 = لا علاقة له بي، 2 = له علاقة بسيطة بي، 3 = له علاقة متوسطة بي، 4 = له علاقة كبيرة بي)

Need to Belong / الحاجة إلى الانتماء

1. When I see on a social network that a friend is somewhere where I wanted to go too, I feel bad / عندما أرى على شبكة اجتماعية أن صديقًا ما في مكان كنت أرغب في الذهاب إليه أيضًا، أشعر بالسوء
2. I get annoyed when my friends do not tag me in posts / أنزعج عندما لا يقوم أصدقائي بالإشارة إلي في المنشورات

3. I get sad to learn from posts that my friends went to events and I wasn't invited / أشعر بالحزن عندما أكتشف من المنشورات أن أصدقائي ذهبوا إلى المناسبات ولم تتم دعوتي

4. Often, I feel sad seeing on social networks that people are happier than I am / غالبًا، أشعر بالحزن عندما أرى على الشبكات الاجتماعية أن الناس أكثر سعادة مني

5. I feel distant from people when I see them happy in posts / أشعر بالبعد عن الناس عندما أراهم سعداء في المنشورات

Need for Popularity / الحاجة إلى الشعبية

1. I get annoyed when my posts do not get many likes and/or comments / أنزعج عندما لا تحصل منشوراتي على العديد من الإعجابات و/أو التعليقات

2. I only post photos or videos that I know my friends will like / أنشر فقط الصور أو مقاطع الفيديو التي أعلم أن أصدقائي سيحبونها

3. I need people to like or comment on my posts / أحتاج إلى أن يقوم الناس بالإعجاب أو التعليق على منشوراتي

4. I am indifferent to my friends' reactions to my posts / لا أبالي بردود فعل أصدقائي على منشوراتي

5. I would like to have more likes and/or comments on my posts / أرغب في الحصول على المزيد من الإعجابات و/أو التعليقات على منشوراتي

Anxiety / القلق

1. I get anxious when my cell phone does not have internet signal / أشعر بالقلق عندما لا يكون لدى هاتفي المحمول إشارة إنترنت

2. If I do not have access to social networks, I think of ways to get connected / إذا لم أتمكن من الوصول إلى الشبكات الاجتماعية، أفكر في طرق للتواصل

3. I think a lot about social networks when I do not have access to them / أفكر كثيرًا في الشبكات الاجتماعية عندما لا أتمكن من الوصول إليها

4. I get restless when I cannot access social networks / أشعر بعدم الارتياح عندما لا أتمكن من الوصول إلى الشبكات الاجتماعية

5. I usually feel irritated by staying disconnected from social networks too long / أشعرعادة بالانزعاج عندما أبقى غير متصل بالشبكات الاجتماعية لفترة طويلة

Addiction / الإدمان

1. When I'm on social networks, I forget my problems / عندما أكون على الشبكات الاجتماعية، أنسى مشاكلي

2. My family and friends complain that I spend a lot of time connected to social networks / تشكو عائلتي وأصدقائي من أنني أقضي الكثير من الوقت متصلًا بالشبكات الاجتماعية

3. When I start checking for updates, I find it hard to leave social networks / عندما أبدأ في التحقق من التحديثات، أجد صعوبة في مغادرة الشبكات الاجتماعية

4. In social situations, I pay more attention to my cell phone than to my friends / في المواقف الاجتماعية، أولي اهتمامًا أكبر لهاتفي المحمول أكثر من أصدقائي

5. I am late to appointments because of social network use / أتأخر عن المواعيد بسبب استخدامي للشبكات الاجتماعية

Rosenberg Self-Esteem Scale / مقياس روزنبرغ لتقدير الذات

Please record the appropriate answer for each item, depending on whether you Strongly agree, agree, disagree, or strongly disagree with it. 1 = Strongly agree 2 = Agree 3 = Disagree 4 = Strongly disagree / يرجى تسجيل الإجابة المناسبة لكل بند، اعتمادًا على ما إذا كنت توافق بشدة، أو توافق، أو لا توافق، أو لا توافق عليها بشدة. 1 = موافق بشدة 2 = موافق 3 = غير موافق 4 = لا توافق بشدة

1. On the whole, I am satisfied with myself / بشكل عام، أنا راضٍ عن نفسي
2. At times I think I am no good at all / أحيانًا أعتقد أنني لست جيدًا على الإطلاق
3. I feel that I have a number of good qualities / أشعر أن لدي عددًا من الصفات الجيدة
4. I am able to do things as well as most other people / أستطيع أن أفعل الأشياء بنفس كفاءة معظم الناس الآخرين
5. I feel I do not have much to be proud of / أشعر أنه ليس لدي الكثير لأفتخر به
6. I certainly feel useless at times / بالتأكيد أشعر أنني عديم الفائدة في بعض الأحيان
7. I feel that I'm a person of worth / أشعر أنني شخص ذو قيمة
8. I wish I could have more respect for myself / أتمنى أن يكون لدي احترام أكبر لنفسي
9. All in all, I am inclined to think that I am a failure / بشكل عام، أميل إلى الاعتقاد بأنني فاشل
10. I take a positive attitude toward myself / أتبنى موقفًا إيجابيًا تجاه نفسي

The World Health Organization-Five Well-Being Index (WHO-5) / WHO-5 معيار التقدم

Please indicate for each of the five statements which is closest to how you have been feeling over the last two weeks. Notice that higher numbers mean better well-being. Example. If you have felt cheerful and in good spirits more than half of the time during the last two weeks, select number three. The response scale is as follows: 0=At no time, 1=Some of the time, 2=Less than half of the time, 3= More than half of the time, 4=Most of the time, 5=All of the time

ضع من فضلك عند كل من الأسئلة الخمسة التالية علامة ضرب في المربع (خانة) التي هي أقرب من كيفما شعرت في الأسبوعين الماضيين. لاحظ بأن درجة أعلى هي لأفضل حالة. مثال: إذا كنت تشعر بأنك كنت سعيدا و بمزاج جيد في أكثر بقليل من نصف الوقت من فترة الأسبوعين الماضيين، فضع علامة ضرب في المربع ذو رقم 3 الموجود في الركن العلوي اليمنى. مقياس الاستجابة كما يلي: 0= بتاتا"، 1= قليل من الوقت (نادرا")، 2= أقل بقليل من الوقت ، 3= أكثر بقليل من الوقت، 4= أكثر الأحيان، 5= دائما"

1. I have felt cheerful and in good spirits /كنت سعيدا" وبمزاج جيد
2. I have felt calm and relaxed / كنت أشعر بالهدوء والاسترخاء
3. I have felt active and vigorous / كنت أشعر بالنشاط والحيوية
4. I woke up feeling fresh and rested /كنت استيقظ نشطا" ومرتاحا"
5. My daily life has been filled with things that interest me /كانت أيامي مليئة بأشياء محببة لنفسي

Thank you for your time and participation.

شكرا لك على وقتك ومشاركتك.
