# Supplementary material for: Development and validation of the Lebanese Social Media Dependency Scale (LSMDS): A cross-sectional study among university students
Source: PLoS One. 2026 Apr 28;21(4):e0344535. doi: 10.1371/journal.pone.0344535 (PMC13123986; doi:10.1371/journal.pone.0344535)

**S1 Appendix B**

| *Factor Loadings* | | | | |
| --- | --- | --- | --- | --- |
|  | Factor 1 | Factor 2 | Factor 3 | Uniqueness |
| SPAI_10 | 0.793 |  |  | 0.395 |
| SPAI_2 | 0.746 |  |  | 0.398 |
| SPAI_3 | 0.744 |  |  | 0.465 |
| SPAI_4 | 0.739 |  |  | 0.352 |
| SPAI_16 | 0.722 |  |  | 0.415 |
| SPAI_19 | 0.7 |  |  | 0.48 |
| SPAI_11 | 0.689 |  |  | 0.476 |
| SPAI_6 | 0.686 |  |  | 0.602 |
| SPAI_5 | 0.679 |  |  | 0.567 |
| SPAI_20 | 0.564 |  |  | 0.61 |
| SPAI_21 | 0.473 |  |  | 0.721 |
| Popularity3 |  | 0.846 |  | 0.28 |
| Popularity1 |  | 0.807 |  | 0.351 |
| Popularity5 |  | 0.767 |  | 0.408 |
| Belongs2 |  | 0.575 |  | 0.615 |
| Popularity2 |  | 0.483 |  | 0.724 |
| Belongs1 |  | 0.447 |  | 0.645 |
| Belongs5 |  | 0.423 |  | 0.654 |
| Anxiety3 |  |  | 0.826 | 0.308 |
| Anxiety4 |  |  | 0.794 | 0.327 |
| Anxiety2 |  |  | 0.736 | 0.433 |
| Anxiety5 |  |  | 0.731 | 0.417 |
| Anxiety1 |  |  | 0.582 | 0.618 |
| SMD_3 |  |  | 0.438 | 0.691 |
| SMD_1 |  |  | 0.355 | 0.82 |
| SMD_2 |  |  | 0.344 | 0.81 |
| *Note.*  Applied rotation method is oblimin. | | | | |

| ***Parallel Analysis*** | | |
| --- | --- | --- |
|  | Real data component eigenvalues | Simulated data mean eigenvalues |
| Factor 1* | 9.551 | 1.445 |
| Factor 2* | 2.745 | 1.385 |
| Factor 3* | 1.547 | 1.329 |
| Factor 4 | 1.12 | 1.277 |
| Factor 5 | 0.974 | 1.235 |
| Factor 6 | 0.87 | 1.198 |
| Factor 7 | 0.756 | 1.169 |
| Factor 8 | 0.723 | 1.142 |
| Factor 9 | 0.709 | 1.107 |
| Factor 10 | 0.634 | 1.078 |
| Factor 11 | 0.622 | 1.05 |
| Factor 12 | 0.579 | 1.027 |
| Factor 13 | 0.549 | 1.002 |
| Factor 14 | 0.523 | 0.972 |
| Factor 15 | 0.483 | 0.943 |
| Factor 16 | 0.466 | 0.92 |
| Factor 17 | 0.426 | 0.895 |
| Factor 18 | 0.393 | 0.866 |
| Factor 19 | 0.368 | 0.84 |
| Factor 20 | 0.336 | 0.813 |
| Factor 21 | 0.329 | 0.788 |
| Factor 22 | 0.289 | 0.764 |
| Factor 23 | 0.268 | 0.737 |
| Factor 24 | 0.26 | 0.706 |
| Factor 25 | 0.256 | 0.678 |
| Factor 26 | 0.224 | 0.634 |
| *Note.*  '*' = Factor should be retained. Results from PC-based parallel analysis. | | |

**Scree plot**


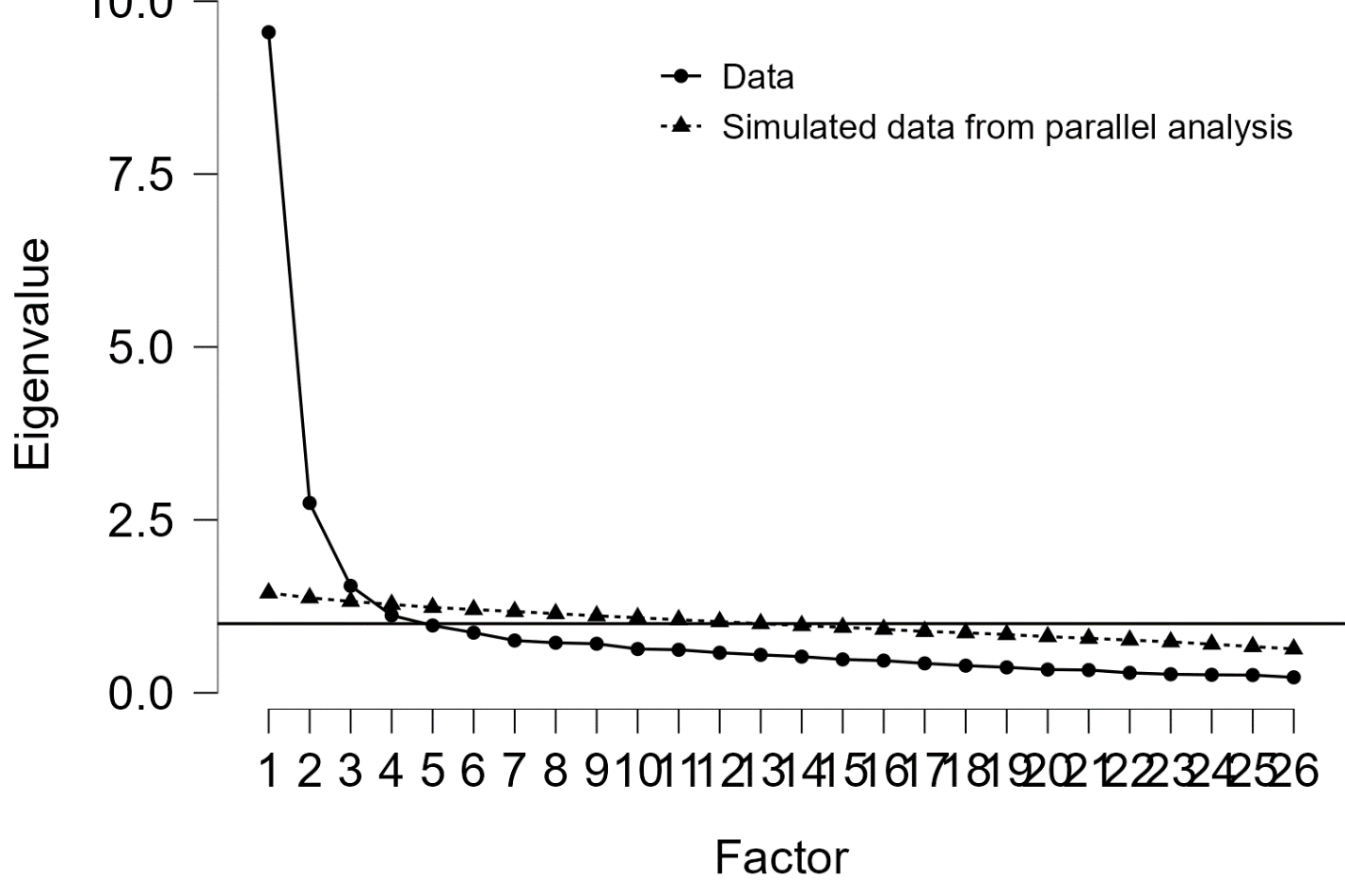

Supplement: S2 Appendix — (DOCX) [file pone.0344535.s002.docx]
